# A case of isolated sinus bradycardia as an unusual presentation of adrenal insufficiency

**DOI:** 10.1016/j.amsu.2021.102727

**Published:** 2021-08-16

**Authors:** Jonathan Vincent M. Reyes, Hafsa Majeed, David Song, Saad Ahmad, Ashley Bray, Talal Almas, Abdulaziz Alshamlan, Joseph J. Lieber

**Affiliations:** a Department of Internal Medicine, Icahn School of Medicine at Mount Sinai Elmhurst Hospital, Queens, NY, USA; b Royal College of Surgeons in Ireland, Dublin, Ireland

**Keywords:** Bradycardia, Adrenal insufficiency, Pacemaker

## Abstract

**Introduction:**

Sinus bradycardia is a common entity encountered in clinical practice. The differential diagnosis is quite broad; it can be an incidental finding in otherwise healthy adults or the first clue to a lethal pathology.

**Case presentation:**

This case highlights a patient who presented with symptomatic sinus bradycardia, which resulted in syncope requiring admission for permanent pacemaker implantation and later found to have an underlying adrenal insufficiency (AI). Patient's underlying hyponatremia was corrected but bradycardia persisted and after the initiation of steroids, bradycardia resolved. Therefore, the likely culprit for bradycardia was AI.

**Discussion:**

Multiple disease processes that manifest with sinus bradycardia are commonly due to the increased vagal tone or the presence of intrinsic conduction disorders. Sinus bradycardia is a common clinical finding with a broad differential including intrinsic and extrinsic causes of sinus node dysfunction or AV block.

**Conclusion:**

It is imperative for clinicians to be aware of rare etiologies for underlying symptomatic bradycardia. While extremely effective at preventing symptomatic bradycardia, avoiding a pacemaker by correcting the underlying etiology of symptomatic bradycardia may improve quality of life and avoid an unnecessary procedure.

## Highlights

• Sinus bradycardia is a common entity encountered in clinical practice. Differential diagnosis is broad; as it can be an incidental finding in otherwise healthy adults or the first clue to a lethal pathology.
• Multiple disease processes manifest with sinus bradycardia commonly due to increased vagal tone (e.g: inferior wall myocardial infarction, drug or toxin exposures, infections, sleep apnea, hormonal and electrolyte disturbances, hypoglycemia, and increased intracranial pressures) or due to intrinsic conduction disorders (e.g sick sinus syndrome, sinus arrest, SA nodal blocks).
• Glucocorticoids have been suggested as having a direct inotropic effect as well as a permissive effect on cardiac myocytes in response to vasoactive peptides like epinephrine and angiotensin II that antagonize the sympathetic nervous system adrenergic receptors.
• It has been proposed that glucocorticoids upregulate expression of ion channels including I_Ks_ (mink, KvLQT1), and I_Kr_ (hERG, MiRP1) which induce outward potassium currents by inducing expression of serum- and glucocorticoid-inducible kinase (SGK1). Lack of glucocorticoids thereby extends the duration of action potential resulting in conduction abnormalities.

## 1 Introduction

Sinus bradycardia is a common entity encountered in clinical practice. The differential diagnosis is often broad; it can be an incidental finding in otherwise healthy adults (e.g: sleep and athletes) or the first clue to a lethal pathology [1,2]. Multiple disease processes manifest with sinus bradycardia commonly due to increased vagal tone (e.g: inferior wall myocardial infarction, drug or toxin exposures, infections, sleep apnea, hormonal and electrolyte disturbances, hypoglycemia, and increased intracranial pressures) or due to intrinsic conduction disorders (e.g sick sinus syndrome, sinus arrest, sinoatrial nodal blocks) [1].

Adrenal insufficiency (AI) is a life-threatening condition resulting in deficient production of glucocorticoids either due to intrinsic adrenal disease or impaired hypothalamic-pituitary axis [3]. The diagnosis of AI is often challenging due to non-specific symptoms including fatigue, weight loss, anorexia, nausea and vomiting, dry skin, loss of libido, muscle and joint pain. An acute major stress with underlying chronic AI or acute adrenal destruction can manifest as a circulatory failure, vasodilatory shock unresponsive to pressors, subsequently resulting in coma and death [4]. Therefore, it is essential to have a high index of suspicion. In addition, this work has been reported in accordance with SCARE [5].

In this case, isolated sinus bradycardia was the first manifestation of AI. The patient was admitted for possible pacemaker implantation due to symptomatic episodes of sinus bradycardia, and lab values revealed hyponatremia. A thorough and systematic evaluation of sinus bradycardia with concurrent hyponatremia led to the diagnosis of AI in this patient.

## 2 Case presentation

**Fig. 1:**
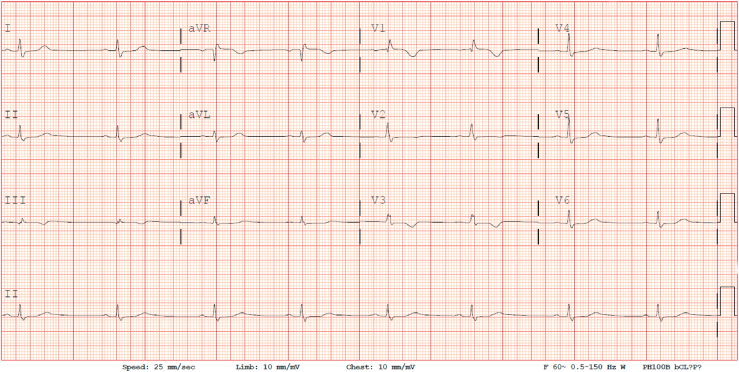
Electrocardiogram revealing sinus bradycardia at the rate of 47 beats per minute with a QRS duration of 125 ms with an incomplete right bundle branch block pattern and PR interval of 200 ms with normal axis and QTc 437 ms.

A 65-year-old male with past medical history significant for hypothyroidism on synthroid 50mcg and hyperlipidemia on lipitor 20 presented with episodes of lightheadedness and dizziness for the past two months. He was having multiple episodes of dizziness in a day, usually at rest. He denied any prodromal symptoms of nausea, diaphoresis, palpitations, chest pain, shortness of breath, or any focal neurologic symptoms. The patient reported no family history of sudden deaths or heart disease. He denied the use of any over-the-counter medications, herbal supplements, and other medications including atrioventricular (AV) nodal blocking agents including beta blockers, calcium channel blockers or digoxin. The patient had no history of alcohol, smoking, or illicit substance usage. He denied any triggers including exercise, change in position, or micturition. He denied any recent fevers, chills, appetite changes, trauma, sick contacts or recent travel. During these episodes, the patient checked his blood pressure (BP) and was found to have blood pressure (BP) in the 70s/50s with heart rate (HR) in the 30s. Electrocardiogram (ECG) was consistent with sinus bradycardia, incomplete right bundle block pattern and PR interval of 200 ms (Fig. 1).

Patient was subsequently found to be hypotensive to 70/50 in the ED not responding to fluids, requiring dopamine drip and was admitted to the cardiac critical care unit for consideration of permanent pacemaker placement for symptomatic bradycardia and close monitoring. To investigate the underlying etiology, transthoracic echocardiogram (TTE) was performed which showed a normal ejection fraction of 60% with mild left ventricular hypertrophy, normal right ventricular contractility, and size. On the physical exam, there was no murmur appreciated otherwise unremarkable except for sinus bradycardia to 50. His labs were notable for sodium of 121 (Ref. 135–145 mEq/L) and hemoglobin was 9.8 g/dl (Ref. 13.5–17.5 g/dl), with MCV of 89 fl (Ref. 80–100 fL), iron 75 (Ref. 60–170 mcg/dl), TIBC 338 (250–450 mcg/dL), ferritin 856 ng/mL (Ref. 20–250 ng/mL); TSH was 5.33 mIU/L (Ref. 0.45–4.12 mU/) with a total T4 5.39 μg/dl (Ref. 5.0–12.0μg/dL). Remaining workup for his hyponatremia revealed urine osmolality of 346, urine sodium of 66 and serum osmolality of 248 consistent with AI or Syndrome of inappropriate antidiuretic hormone secretion (SIADH). Endocrine was consulted and a cosyntropin stimulation test was performed. Results of cosyntropin stimulation test revealed: cortisol baseline 4 μg/dL, after 30 minutes 8 μg/dL, after 60 minutes 9 μg/dL. Of note, a standard cosyntropin stimulation test, which tests the ability of adrenocorticotropic hormone (ACTH) to stimulate the release of cortisol, in the setting of intact adrenal function usually yields a post-ACTH cortisol of ≥18 μg/dl. Given the results demonstrating acute adrenal insufficiency, the patient was started on intravenous (IV) hydrocortisone 25 mg twice daily. Given ACTH levels were not measured, we were unable to differentiate between primary and chronic secondary disease processes.

However, the patient was still bradycardic to the 50s despite correction of hyponatremia to 136, which is a common cause of hyponatremia [6]. Patient was continued with IV hydrocortisone for the next few days and bradycardia resolved with a rate of 65. As a result, the decision was made to postpone the pacemaker implantation. His symptoms completely resolved after receiving steroids; therefore the diagnosis of sinus bradycardia secondary to adrenal insufficiency was made in the absence of hyponatremia. The patient was later evaluated in the cardiology clinic for follow-up 1-month post-discharge with no interval symptoms. He was placed on a 14 day Holter monitor for surveillance which did show any further episodes of bradycardia.

## 3 Discussion

Sinus bradycardia is a common clinical finding with a broad differential including intrinsic and extrinsic causes of sinus node dysfunction or AV block. A thorough history to elicit precipitating factors, nocturnal symptoms, recent surgery, travel history,a complete review of systems, list of medications and basic lab tests including thyroid function tests can aid in narrowing the differential [2]. The patient's symptoms were not related to exercise, therefore it was unlikely to be chronotropic incompetence [7]. His chest radiograph did not show any hilar lymphadenopathy and his review of systems were unremarkable; therefore, sarcoidosis or other autoimmune diseases were deemed unlikely [8]. The patient also had not reported any recent travel or tick bites therefore, Lyme disease was low on the differential [9]. The patient did not have any risk factors such as obesity, high waist circumference, snoring at night time or daytime sleepiness for sleep apnea therefore sleep apnea was ruled out [10]. He was not on any AV nodal blocking medications that are known to cause sinus bradycardia. He had an unremarkable echocardiogram therefore infiltrative diseases of the heart or infectious endocarditis were ruled out [11]. The only abnormal finding in his labs was hyponatremia which was a clue to his diagnosis. However, hyponatremia as a cause of the bradycardia was ruled out once hyponatremia was corrected, and bradycardia persisted. In addition, due to the resolution of symptoms after starting steroids, intrinsic disorders of conduction were deemed unlikely.

The pathophysiology of cardiac manifestations of AI is not completely understood [12]. Glucocorticoids have a permissive effect on cardiac myocytes in response to vasoactive peptides like epinephrine and angiotensin II that antagonize the sympathetic nervous system adrenergic receptors [13]. Moreover, some studies suggest a direct inotropic effect of glucocorticoids [14]. Additional mechanisms include stimulation of epinephrine synthesis in the adrenal medulla, inhibition of catechol-O-methyltransferase, an enzyme that inactivates catecholamines, maintenance of membrane calcium transport function in cardiac sarcoplasmic reticulum, and [15,16] cortisol mediated transcription and expression of α1 -adrenergic receptors in cardiac myocytes [17].

Prior studies have reported various ECG abnormalities in adrenal insufficiency, most common abnormalities are flat or inverted T-waves, prolonged QT interval, prolonged PR or QRS interval, depressed ST segment, and low voltage [18]. It has been proposed that glucocorticoids upregulate the expression of ion channels including I_Ks_ (mink, KvLQT1), and I_Kr_ (hERG, MiRP1) which induce outward potassium currents by inducing expression of serum- and glucocorticoid-inducible kinase (SGK1) [19]. Lack of glucocorticoids as a result extends the duration of action potential resulting in conduction abnormalities. Although rare, isolated cardiac rhythm abnormalities including cardiac arrest, torsade de pointes due to QT prolongation, and sinus bradycardia due to AI have been reported [20]. In all the case reports, treatment included steroid replacement typically with hydrocortisone which resolved these arrhythmias. Hydrocortisone is the treatment of choice in acute AI due to its mineralocorticoid activity, starting with a dose of IV 15–25mg in two or three divided doses per day, with the highest dose given in the morning with the next dose in the afternoon in an attempt to reproduce the circadian rhythm of cortisol secretion [21]. Later, glucocorticoid therapy is tapered to the minimum effective dose to avoid complications of over-replacement.

## 4 Conclusion

Sinus bradycardia is a common reason for referral to a cardiologist for placement of permanent pacemaker implantation. It is imperative for clinicians to be aware of rare etiologies for underlying symptomatic bradycardia. While extremely effective at preventing symptomatic bradycardia, avoiding a pacemaker by correcting the underlying etiology of symptomatic bradycardia may improve quality of life and avoid an unnecessary procedure. The ability of clinicians to address the underlying cause of symptomatic bradycardia may reduce hospitalizations for recurrent symptoms.

## Provenance and peer review

Not commissioned, externally peer-reviewed.

## Consent

Written informed consent was obtained from the patient for publication of this case report and accompanying images. A copy of the written consent is available for review by the Editor-in-Chief of this journal on request.

## Ethical approval

Obtained.

## Sources of funding

None.

## Author contribution

JR, HM wrote the abstract, introduction, case, discussion, conclusion.

AB, DS, TA, JL performed critical edits and final revision, figures.

## Registration of research studies

Name of the registry: NA.

Unique Identifying number or registration ID: NA.

Hyperlink to your specific registration (must be publicly accessible and will be checked): NA.

## Guarantor

Talal Almas.

RCSI University of Medicine and Health Sciences.

123 St. Stephen's Green Dublin 2, Ireland.

Talalamas.almas@gmail.com.

+353834212442.

## Declaration of competing interest

None.
